# Melanoma immunotherapy

**DOI:** 10.18632/oncoscience.228

**Published:** 2015-08-31

**Authors:** Abhishek D. Garg, Aleksandra M. Dudek-Peric, Patrizia Agostinis

**Affiliations:** Cell Death Research & Therapy (CDRT) Lab, Department of Cellular Molecular Medicine, KU Leuven University of Leuven, Belgium

**Keywords:** immunogenicity, immunogenic cell death, calreticulin, immune-checkpoint blockade, patient treatment

Cutaneous malignant melanoma is amongst the most aggressive cancer types, which typifies the paradox of being simultaneously highly antigenic and highly immunoevasive [1, 2]. However, various anti-melanoma immunotherapies have shown limited clinical success e.g. IL2, IFN-α2a or dendritic cells (DCs)-based vaccines [1–3]. Subsequent research showed that tumor-induced immunosuppression caused failure of above mentioned immunotherapies and thus overcoming this was proposed to be the key to success. This assumption proved correct, as evident by the impressive anti-melanoma clinical responses achieved with anti-CTLA4/PD-1/PD-L1 antibodies (i.e. immune-checkpoint inhibitors or ICIs) [2, 3]. However, despite this success, there remains a sizeable subset of melanoma patients that don't respond to ICI-therapies [3]. While efforts are underway to dig out new immunological therapeutic targets yet recent research suggests that cancer cell-autonomous events can also play an important role in resistance to immunotherapies. For instance, recently melanoma cells were reported to mount resistance against anti-CTLA4 immunotherapy by up-regulating surface PD-L1 and thereby causing T cell exhaustion (Figure 1) [2]. Similarly, melanoma cell-autonomous WNT/β-catenin signaling was found to cause T cell exclusion from tumor microenvironment thereby creating ICI-therapy resistance (Figure 1) [3].

**Figure 1:**
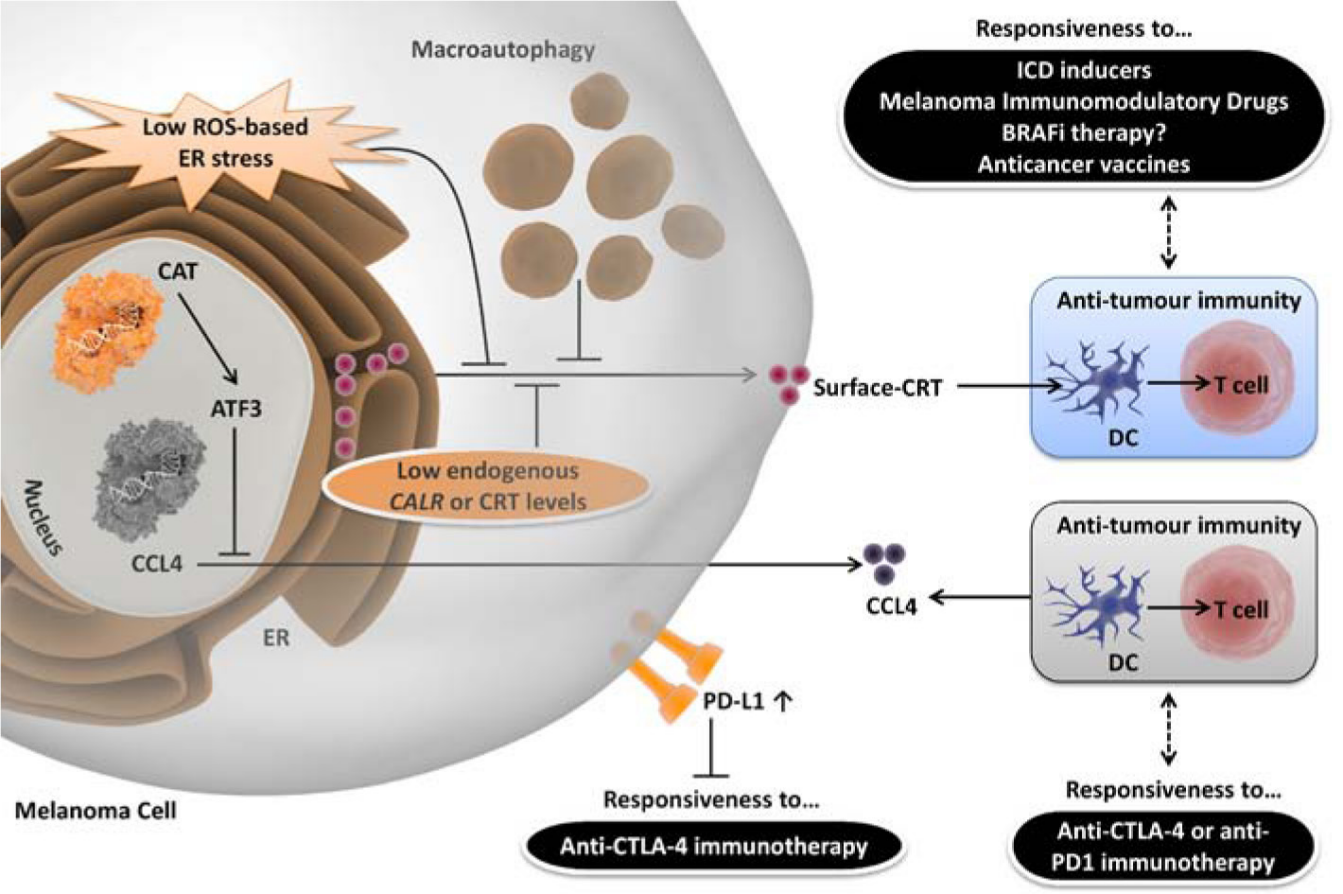
Schematic representation of melanoma cell-autonomous resistance mechanisms against immunotherapeutic paradigms Melanoma cell-autonomous resistance can be posed to ICI-drugs (anti-CTLA4/anti-PD1 immunotherapy) either via increased surface PD-L1 levels or through WNT/β-catenin (CAT) signaling-based increase in ATF3 levels, which in turn reduce the T cell-attracting chemokine, CCL4. On the other hand, resistance against the effect of ICD, vaccines or immunomodulatory chemotherapeutics/drugs (melphalan/BRAFi) can operate on the level of low surface-CRT. Low ROS-based ER stress, high macroautophagy and/or low endogenous CRT/CALR levels can all contribute towards decreased surface-CRT.

The above research on cancer cell-autonomous resistance has largely focused on ICI-therapies. However, it is also widely recognized that a durable “resetting” of the tumor microenvironment towards immune-susceptibility requires effective treatment with cytotoxic drugs that activate anticancer immunity [4–6]. We believe that the highest degree of such “reset” is achievable by inducers of immunogenic cell death (ICD) [5, 6]. ICD is a cell death routine triggered by a limited set of assorted anticancer therapies (anthracyclines, radiotherapy, photodynamic therapy/PDT, oncolytic viruses), that is accompanied by danger signaling-driven, spatiotemporally defined, surface exposure or secretion/release of damage-associated molecular patterns (DAMPs) like surface-calreticulin (ecto-CRT, a quintessential ‘eat me’ signal that mediates immunogenicity) [4–6]. These DAMPs than interact with their cognate receptors, and help in instigating a potent anti-tumor immune response that helps eradicate residual cancer cells [4, 5]. ICD has shown significant preclinical promise in a number of experimental models and some clinical promise in patients of lung cancer, breast cancer, ovarian cancer and lymphoma [4–6].

Remarkably, melanoma, which shows palpable pre-clinical susceptibility to ICD, has usually failed to show clinical responsiveness to single-agent ICD inducers like, anthracyclines/doxorubicin [7]. These paradoxical results have driven some of our recent research endeavors aimed at studying the ICD-melanoma link. We have found that melanoma cell-autonomous resistance to ICD can operate on two major levels i.e. macroautophagy activity [6, 8] (Figure 1) and general capability of surface-exposing CRT [1, 4] (Figure 1), as further discussed below.

## Macroautophagy activity

Using a *bona fide* ICD inducer, Hypericin-based PDT (Hyp-PDT) [6], we found that human melanoma cell-associated macroautophagy suppresses exposure of ecto-CRT (possibly by reducing oxidative-ER stress), which in turn reduces maturation of the interacting DCs, DC-derived IL6 production and proliferation of IFN-γ producing CD4^+^/CD8^+^ T cells (Figure 1) [6]. Thus, these results unraveled a role for ROS-induced autophagy in weakening the functional interaction between dying melanoma cells and immune cells [6]. These results were recently, partially, extended to BRAF^V600E^ inhibitor-resistant melanoma cells where autophagy was shown to suppress exposure of ecto-CRT and ecto-HSP90 [8] (Figure 1). Nevertheless, it remains to be seen whether this activity of cell-associated autophagy also weakens the interactions between immune cells and BRAF^V600E^ inhibitor-resistant melanoma cells [8].

## General capability of surface-exposing CRT

In a recent report we demonstrated the existence of a broad ICD-resistance mechanism using an AY27 rat bladder cancer model and two *bona fide* ICD inducers (mitoxantrone and Hyp-PDT) [4]. This ICD-resistant phenotype stemmed from low endogenous CRT protein levels in cancer cells (i.e. CRT^low^-phenotype) which resulted in defective ecto-CRT levels (Figure 1), which further caused severely reduced phagocytic clearance of treated cancer cells, which ultimately lead to the failure of tumor-rejecting immunity [4]. Interestingly, we found that a subset of cancer patients of various cancer-types tend to exhibit *CALR*^low^ or CRT^low^-tumors [4]. Moreover, we observed that tumoral *CALR*^high^-phenotype was predictive of positive clinical responses to therapy with ICD inducers like radiotherapy or paclitaxel in non-small cell lung or ovarian cancer patients, respectively (but not non-ICD inducer like topotecan in ovarian cancer) [4]. Additionally, tumoral *CALR* levels positively correlated with the levels of genes relevant for phagosome maturation or processing in only the clinical ICD set-up [4] (Figure 1). Importantly, we found that a subset of melanoma patients also had the tendency to show CRT^low^-tumors thereby hinting at the possible existence of above resistance mechanism in melanoma [4]. Possibility of such resistance mechanism in melanoma is of high implication since our research has found ecto-CRT to be crucial for immunogenicity of dying melanoma cells [1]. More specifically, we have shown that a well-established anti-melanoma chemotherapeutic, melphalan fails to induce sufficiently high immunogenicity *in vivo*, because it is not able to induce the relevant threshold levels of reactive oxygen species (ROS)-based ER stress required for ecto-CRT induction (Figure 1) [1]. Ecto-CRT on melphalan-treated melanoma cells was “restored” when the low ROS-based ER stress was increased by combining with the ER stressor, thapsigargin [1]. Importantly, we also observed that dying melanoma cells were largely reliant on ecto-CRT for immunogenicity since ecto-HSP90, which was emphatically exposed by melphalan-treatment failed to mediate immunogenicity [1]. In near future, it would be interesting to find whether overall CRT or *CALR* levels in melanoma are predictive of clinical responses to ICD or immunotherapy and/or regulate overall levels/spatial distribution of CD8^+^ T cell-infiltrates.

In conclusion, there clearly exist melanoma cell-autonomous mechanisms that disrupt responses to immunotherapy with ICI-drugs or ICD-inducers. However, a future exome-sequencing or deep-sequencing study utilizing melanoma patient samples is required to characterize melanoma genotypes that associate with poor T cell infiltration and/or clinical responses to anti-melanoma therapeutics.
